# Editorial: New approach methods in immunology

**DOI:** 10.3389/fimmu.2024.1488534

**Published:** 2024-09-18

**Authors:** Thomas Hartung, Jeffrey John Bajramovic, Susan Gibbs, Emanuela Corsini

**Affiliations:** ^1^ Center for Alternatives to Animal Testing (CAAT), Doerenkamp-Zbinden-Chair for Evidence-based Toxicology, Johns Hopkins Bloomberg School of Public Health, Baltimore, MD, United States; ^2^ Center for Alternatives to Animal Testing (CAAT)-Europe, University of Konstanz, Konstanz, Germany; ^3^ 3Rs Centre Utrecht, Utrecht University, Utrecht, Netherlands; ^4^ Department of Molecular Cell Biology and Immunology, Amsterdam University Medical Center (UMC), Infection & Immunity Institute, Vrije Universiteit Amsterdam, Amsterdam, Netherlands; ^5^ Department of Oral Cell Biology, Academic Centre for Dentistry Amsterdam (ACTA), University of Amsterdam and Vrije Universiteit, Amsterdam, Netherlands; ^6^ Department of Pharmacological and Biomolecular Sciences ‘Rodolfo Paoletti’, Università degli Studi di Milano, Milan, Italy

**Keywords:** alternatives to animal testing, new approach methods (NAM), microphysiological system (MPS), artificial intelligence, cell culture

The field of immunology is undergoing a significant transformation with the advent of new approach methods (NAM) as alternatives to traditional animal models. These advancements are driven by economic, ethical, and scientific motivations, particularly the need to bridge the translational gap between animal studies and human clinical trials, often referred to as the ‘valley of death.’

Immunology is uniquely positioned compared to other fields with respect to NAMs due to the intricate complexity and dynamic nature of the immune system. Unlike other biological systems, the immune system involves a vast array of cell types, signaling molecules, and interactions that span different tissues and organs. Key aspects that make immunology special in the context of NAMs include:

1. Complexity of Interactions: Immunological responses involve a highly coordinated interplay between innate and adaptive immune cells, which must be accurately replicated to understand mechanisms of disease and therapeutic interventions.
2. Spatial and Temporal Dynamics: Immune responses are characterized by precise spatial and temporal dynamics, requiring advanced modeling techniques to simulate processes such as cell migration, tissue infiltration, and the sequential activation of immune pathways.
3. Genetic and Functional Variability: The immune system exhibits significant genetic and functional variability among individuals, necessitating NAMs that can capture this diversity to provide more personalized insights into immune responses and disease susceptibilities.
4. Involvement of Multiple Organs and Systems: Immunological studies must consider the interconnectedness of various organs and systems, such as the lymph nodes, spleen, bone marrow, and mucosal surfaces, making the development of comprehensive NAMs particularly challenging and crucial.
5. Impact of Environmental Factors: The immune system is highly responsive to environmental factors, including pathogens, toxins, and lifestyle influences. NAMs must incorporate these variables to fully understand their impact on immune function and disease progression.

Traditional animal models often fail to replicate the human immune system’s complexity accurately, leading to translational gaps. 90 million years of evolution under enormous selective pressure since rodents split from humans have left their mark. NAMs offer a more ethical and potentially more accurate alternative, enhancing the relevance of immunological research to human health. Especially animal studies involving infections to challenge the immune system are often involving very severe suffering.

Overall, the special requirements and challenges of immunology drive the need for sophisticated and innovative NAMs that can capture the multifaceted nature of immune responses, offering more relevant and humane alternatives to traditional animal-based research. This editorial summarizes the state-of-the-art developments in NAM for immunology, highlighting the efforts to mimic *in vivo* biology accurately and overcome specific challenges inherent to immunological research.

The 18 accepted contributions showcase advances in immunological non-animal methods:

Ahimbisibwe et al. examine the feasibility of conducting postmortem studies for tuberculosis research in Uganda. The researchers found good acceptance from next-of-kin for tissue donation, and demonstrated that postmortem procedures and tissue processing could be completed within 8 hours of death while maintaining cell viability for up to 14 hours. This work establishes the feasibility of using postmortem tissues for immunological studies, providing a valuable resource for understanding tissue-specific immune responses in humans as a valuable tool for understanding tissue-specific immune responses in tuberculosis and other diseases.

Arlat et al. present a method for generating functional adipose tissue-resident macrophages using 3D culture of stromal vascular cells from adipose tissue. The researchers show that these macrophages have distinct characteristics from bone marrow-derived macrophages, including metabolic activity and polarization responses. Importantly, single-cell analysis indicates the cultured macrophages mirror phenotypic and functional traits of *in vivo* adipose tissue-resident macrophages. This technique provides a valuable tool for studying adipose tissue macrophage biology without the need for cell sorting.

Brun et al. detail a 24-color flow cytometry panel that allows comprehensive immunophenotyping of human peripheral blood cells, facilitating high-throughput screening of chemicals and identifying affected cell types and signaling pathways. This review examines research on Ca^2+^ signaling and inflammation in peripheral blood mononuclear cells (PBMCs) through bibliometric analysis. The authors found that while Ca^2+^ signaling is crucial for immune cell function, flow cytometry-based analysis of Ca^2+^ in PBMCs is still underdeveloped. They highlight knowledge gaps regarding intracellular Ca^2+^ players in PBMCs and propose flow cytometry as a complementary method to microscopy for studying Ca^2+^ dynamics in these cells.

Ehlers et al. developed a scalable microfluidic platform for modeling vascular inflammation using human endothelial cells. The system enables real-time measurement of endothelial barrier function via transendothelial electrical resistance (TEER) in 64 parallel microfluidic channels. The authors demonstrated the platform can detect inflammatory responses to cytokines and immune cells, including changes in barrier function, adhesion molecule expression, and immune cell migration. They propose it as a powerful tool for studying vascular inflammation and screening anti-inflammatory drugs.

Hölken et al. introduce a 3D full-thickness skin model with dermal dendritic cell surrogates derived from THP-1 cells, this model effectively mimics immune responses to sensitizers and serves as a valuable tool for studying skin sensitization and inflammatory responses. The integrated dendritic cells remained functional and responded to sensitizers by upregulating maturation markers. The model could detect suppression of dendritic cell activation by dexamethasone treatment. This immune-competent skin model may be useful for testing potential sensitizers and anti-inflammatory compounds.

Hoonakker et al. examine the *in vitro* toxicity testing of *Clostridium perfringens* type C beta-toxin using a cell-based assay with THP-1 cells. The researchers developed and validated the assay as an alternative to mouse-based tests for toxicity assessment of veterinary vaccines. They show the assay can detect toxin activity at high dilutions and is suitable for testing at multiple stages of vaccine production. The authors suggest this approach could reduce animal use in toxicity testing for *C. perfringens* vaccines.

Korkmaz et al. describe an *in silico* mechanistic modeling approach is used to investigate the dynamics of the immune response following burn injuries. The model uses agent-based techniques to simulate the behavior of inflammatory agents, providing insights into post-burn inflammation and potential therapeutic interventions. The model incorporates various immune cells and cytokines to capture the complex dynamics of inflammation. Simulations identified key factors influencing inflammation intensity, including endothelial cell count. This computational approach could help predict inflammatory responses and test potential interventions for burn injuries.

Li et al. review recent developments in phage display-based nano immunosensors for detecting cholera toxin. The authors discuss how engineered bacteriophages with specific antibody fragments or mimotopes can enable sensitive and precise toxin detection. This approach offers an ethical alternative to animal-derived methods and has the potential to significantly improve cholera toxin detection capabilities. The authors highlight how engineered bacteriophages with specific antibody fragments or mimotopes can enable precise and sensitive detection of cholera toxin. This approach offers a promising alternative to animal-derived methods, potentially transforming cholera toxin detection with improved safety, sensitivity and ethics.

Lu et al. developed an *in vitro* assay using the DC2.4 dendritic cell line to evaluate the immunogenicity of peptide-based vaccines. The assay measures dendritic cell uptake, maturation, and cytokine production in response to vaccine candidates. It showed good correlation with previously reported *in vivo* results for various peptide constructs. This assay could serve as a useful tool for screening vaccine candidates before animal testing.

Matsuba et al. developed a method to generate dendritic cells (DCs) from mouse bone marrow using small molecule inhibitors. The cocktail of inhibitors called YPPP enhanced DC maturation, increased IL-12 production, and improved T cell activation compared to conventional methods. In tumor models, YPPP-derived DCs showed enhanced anti-tumor effects, especially when combined with anti-PD-1 therapy. The authors propose this as a useful method for generating DCs with improved immunostimulatory functions for cancer immunotherapy.

Morrison et al. review multi-organ-on-chip (multi-OoC) systems for studying immunotoxicity. These systems replicate systemic immunological processes by integrating various immune cells and tissues in a controlled *in vitro* environment to study autoimmune diseases and immune responses. The authors describe various models for organs like lung, skin, intestine, and lymphoid tissues, highlighting how they recapitulate key aspects of organ-specific immunity. While progress has been made, especially for innate immunity, the authors note that incorporating adaptive immunity remains challenging. They suggest organ-on-chip models will be valuable for studying disease mechanisms and drug effects, but further development is needed to fully replicate systemic immune responses.

Mulder et al. developed a human full skin equivalent burn wound model based on the collagen-elastin matrix MatriDerm^®^ incorporating peripheral blood-derived monocytes and T cells to study thermal injuries and the post-burn inflammatory processes. This study marks a significant step forward in creating an immunocompetent human skin model for investigating burn-induced immune responses, examining alterations in marker expression on immune cells and the secretion of cytokines in the culture medium.

Pierzchalski et al. present a suite of flow cytometry-based assays utilizing primary human blood cells to test the immunotoxic effects of chemicals. These assays target various immune cells, including T cells, NK cells, and B cells, offering a robust alternative to traditional cell line-based assays. The assays cover activation of various immune cell types including T cells, NK cells, B cells, basophils, and innate-like T cells. The authors demonstrate the assays can detect immunomodulatory effects of chemicals and propose them as a valuable tool for *in vitro* toxicity and immunomodulatory testing. The assays use primary human cells and provide high-throughput, multiparametric analysis. This test battery provides a standardized approach for evaluating chemical impacts on human immune cell function that could support toxicity testing and drug development.

Sun et al. proposed a novel train of thought, “integration”, to augment the breadth and depth of bioinformatics in peptide-based therapeutics. The advancement of epitope-based vaccines is a significant breakthrough in bioinformatics research, making the exploration of pathogen epitopes more convenient and cost-effective.

Ugolkov et al. review mathematical models of autoimmune diseases that focus on mechanistic descriptions of the immune system. The authors identified 38 models covering 13 autoimmune conditions, with most using systems of ordinary differential equations to model immune components and interactions. While the models provide insights into autoimmune processes, many lack rigorous validation against clinical data or incorporation of pharmacokinetic elements. The authors suggest a need for more robust quantitative systems pharmacology models to support drug development for autoimmune diseases.

Yan et al. developed a novel sandwich ELISA method to detect the peanut allergen Ara h 2 and measure changes in its immunoreactivity in processed foods. The assay uses antibodies specific to IgE epitopes of Ara h 2 and shows high sensitivity and specificity. It was able to detect Ara h 2 in various food samples and measure changes in immunoreactivity after processing. This method could be useful for monitoring allergen levels and potential allergenicity of peanut proteins in foods.

Yang et al. analyze single-cell RNA sequencing data to create a cell atlas of the human thymus and investigate changes during aging. The researchers identified key signaling pathways and transcriptional regulators involved in early thymocyte development. They found that IGFBP5 expression increases with age in thymic epithelial cells and may play a role in thymic involution. The findings provide new insights into thymus biology and age-related changes.

Zaderer et al. This study compares the pathogenicity of Delta and Omicron SARS-CoV-2 subvariants using a human 3D respiratory model. Findings show that Delta penetrates deep into respiratory tissues causing significant damage and inflammation, while Omicron subvariants remain superficial, causing less tissue damage and lower inflammatory responses. These findings provide insights into the different clinical presentations of Delta and Omicron infections and highlight the importance of early virus-tissue interactions in shaping disease severity.

The collective efforts reflected in these articles demonstrate significant progress in the development and application of NAM in immunology. By continuing to refine these methods and overcome existing challenges, the scientific community can advance towards more ethical, cost-effective, and human-relevant research methodologies.

